# Viral envelope proteins fused to multiple distinct fluorescent reporters to probe receptor binding

**DOI:** 10.1002/pro.4974

**Published:** 2024-03-27

**Authors:** Ilhan Tomris, Roosmarijn van der Woude, Rebeca de Paiva Froes Rocha, Alba Torrents de la Peña, Andrew B. Ward, Robert P. de Vries

**Affiliations:** ^1^ Department of Chemical Biology & Drug Discovery, Utrecht Institute for Pharmaceutical Sciences Utrecht University The Netherlands; ^2^ Department of Integrative Structural and Computational Biology The Scripps Research Institute La Jolla California USA

**Keywords:** attachment protein, GFP, hemagglutinin, influenza a virus, multivalency, receptor‐binding, SARS‐CoV‐2

## Abstract

Enveloped viruses carry one or multiple proteins with receptor‐binding functionalities. Functional receptors can be glycans, proteinaceous, or both; therefore, recombinant protein approaches are instrumental in attaining new insights regarding viral envelope protein receptor‐binding properties. Visualizing and measuring receptor binding typically entails antibody detection or direct labeling, whereas direct fluorescent fusions are attractive tools in molecular biology. Here, we report a suite of distinct fluorescent fusions, both N‐ and C‐terminal, for influenza A virus hemagglutinins and SARS‐CoV‐2 spike RBD. The proteins contained three or six fluorescent protein barrels and were applied directly to cells to assess receptor binding properties.

## 1 INTRODUCTION

The surfaces of influenza A and SARS‐CoV‐2 viruses are decorated with glycoproteins that mediate receptor binding. For influenza A, hemagglutinin (HA), a homotrimer type 1 transmembrane glycoprotein, mediates receptor binding through interaction with α2‐3 or α2‐6‐linked sialylated glycan structures on host cells (Wu & Wilson, 2020; Liao et al., 2010; Ji et al., 2017; Gambaryan et al., 2005). Avian H3 and H5 viruses recognize only α2‐3 linked sialic acid (SA), preferably on branched N‐ and O‐glycans (Gambaryan et al., 2005; Stevens et al., 2006; Peng et al., 2017). Human H1 and H3 display specificity to Neu5Acα2‐6Gal (α2‐6‐linked SA), albeit with bias to certain features, such as contemporary H3 recognizing longer oligosaccharides with poly‐LacNac repeats (Peng et al., 2017; Broszeit et al., 2021; Byrd‐Leotis et al., 2019; Spruit et al., 2023; Gulati et al., 2013), whereas seasonal H1 shows preference to shorter structures (Liao et al., 2010; Stevens et al., 2006; Peng et al., 2017; Nemanichvili et al., 2019).

For SARS‐CoV‐2, the S spike glycoprotein mediates host‐cell receptor interaction using its receptor binding domain (RBD) (Walls et al., 2020; Wang et al., 2020). The S spike glycoprotein is composed of an S1 and S2 subunit; the RBD is a stretch of amino acids located within the C‐terminal domain (CTD) of the S1 subunit and directly interacts with the angiotensin‐converting enzyme 2 (ACE2) receptor. Hereafter, proteolytic cleavage by TMPRSS2 primes the S2 subunit, enabling membrane fusion and viral entry into host cells (Fraser et al., 2022). Other human and animal coronaviruses, such as NL63, TGEV, PRCV, MERS, and SARS, also utilize the RBD located in the CTD for receptor binding (Everest et al., 2022). Furthermore, many coronaviruses (TGEV, BCoV, MERS, HKU1, and OC43) possess an evolutionarily conserved glycan‐binding region located in the N‐terminal domain of the S1 subunit, similarly shown for SARS‐CoV‐2 (Qing et al., 2020; Tomris et al., 2023; Li, 2015). For most influenza A and Coronavirus strains, finer receptor binding specificities are not entirely characterized; therefore, straightforward tools to study receptor binding properties would augment future studies (Nemanichvili et al., 2019; Tomris et al., 2023; Bouwman et al., 2021; Nguyen et al., 2022; van der Woude et al., 2020; Liu et al., 2021; Heesters et al., 2021; Nemanichvili et al., 2022).

**FIGURE 1:**
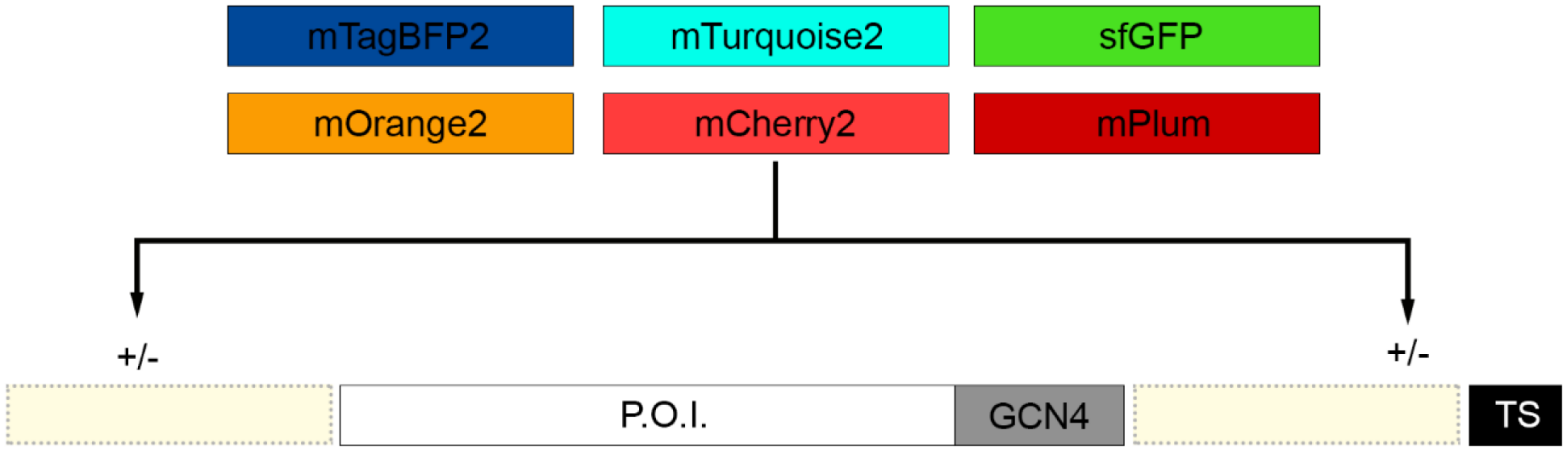
Improved palette of fluorescent proteins covering the visible spectrum. Fluorescent probes mTagBFP2, mTurquoise2, sfGFP, mOrange2, mCherry2, mPlum were cloned upstream (N‐terminal) and/or downstream (C‐terminal) of the protein of interest (P.O.I., HA and SARS‐CoV‐2 RBD). These FFP possess distinct excitation (ex) and emission (em) properties: mTagBFP2 (ex: 399, em: 454), mTurquoise2 (ex: 434, em: 474), sfGFP (ex: 485, em: 510), mOrange2 (ex: 549, em: 565), mCherry2 (ex: 589, em: 610) and mPlum (ex: 590, em: 649).

The discovery of the naturally occurring green fluorescent protein (GFP) derived from Aequorea Victoria has enabled the characterization, visualization, and localization of proteins within cellular processes (Chudakov et al., 2010; Tsien, 1998; Katz et al., 1998; Kneen et al., 1998; Hanson & Kohler, 2001). The fusion of GFP to proteins of interest (P.O.I.) with molecular engineering enables the expression in a 1:1 ratio, allowing quantitative approaches for functional characterization (Kremers et al., 2011). Besides the well‐known GFP, multiple spectral variants have been developed, spanning a wide range of visible spectrum (www.fpbase.org), enabling multiparameter measurements or advanced microscopy techniques (Sekar & Periasamy, 2003; Bastiaens & Squire, 1999; Bacia et al., 2006; O'Donnell et al., 2013). Furthermore, recombinant expression of viral proteins fused to fluorescent proteins would elucidate protein functionality, for example, receptor‐binding. Henceforth, we designed and expressed recombinant fluorescent HA and RBD proteins that allow for direct observation of receptor‐binding interactions using advanced imaging techniques. The placement of a fluorescent protein concerning a P.O.I. may influence functionality, such as protein folding and interference of target/binding domains (Snapp, 2005). Cytoplasmic proteins tolerate FP fusions at the NH_2_‐ (N‐terminal) or COOH‐terminus (C‐terminal) since the N‐ and C‐terminus are exposed on the surface of the protein rather than being buried (Hovmoller & Zhou, 2004). For transmembrane (glyco)proteins, the placement of the fluorescent protein can be restrictive towards expression and functionality (Kermani, 2024). HA proteins from influenza A viruses are membrane‐bound, with the stem (HA2) anchoring the HA in the membrane using the transmembrane domain (Chang et al., 2008). We have previously shown that truncation of HA2 enables and tolerates C‐terminal fusion of superfolder GFP (sfGFP) (Nemanichvili et al., 2019), with N‐terminal fusions also being tolerated in our expression system using neuraminidase (van der Woude et al., 2020). Following translation, the N‐terminus is close to the C‐terminus on the viral membrane for a well‐folded HA, indicating potential tolerance for a recombinantly expressed fluorescent fusion protein (FFP) without interfering with HA1 receptor‐binding properties. Recombinant HAs were created with a GCN4 trimerization domain, Strep‐Tag (TwinStrep, TS), with or without an sfGFP at the N‐ and C‐terminus (Figure 1). Additionally, we also generated recombinant HA with an improved palette of fluorescent proteins (mTagBFP2, mTurquoise2, mOrange2, mCherry2, mPlum) with varying spectral properties (Table S1). This enables multiparameter measurements at different wavelengths using HAs with distinct receptor binding properties with advanced imaging techniques, such as FRET and flow cytometry. Furthermore, in previous studies, expression yields were enhanced by genetic fusions of GFP (van der Woude et al., 2020; Rana et al., 2018); therefore, we also characterized the influence on expression yields of genetic fusions with distinct fluorescent proteins.

## 2 RESULTS

### 2.1 Genetic fusions of an improved fluorescent palette with influenza HA proteins

**FIGURE 2:**
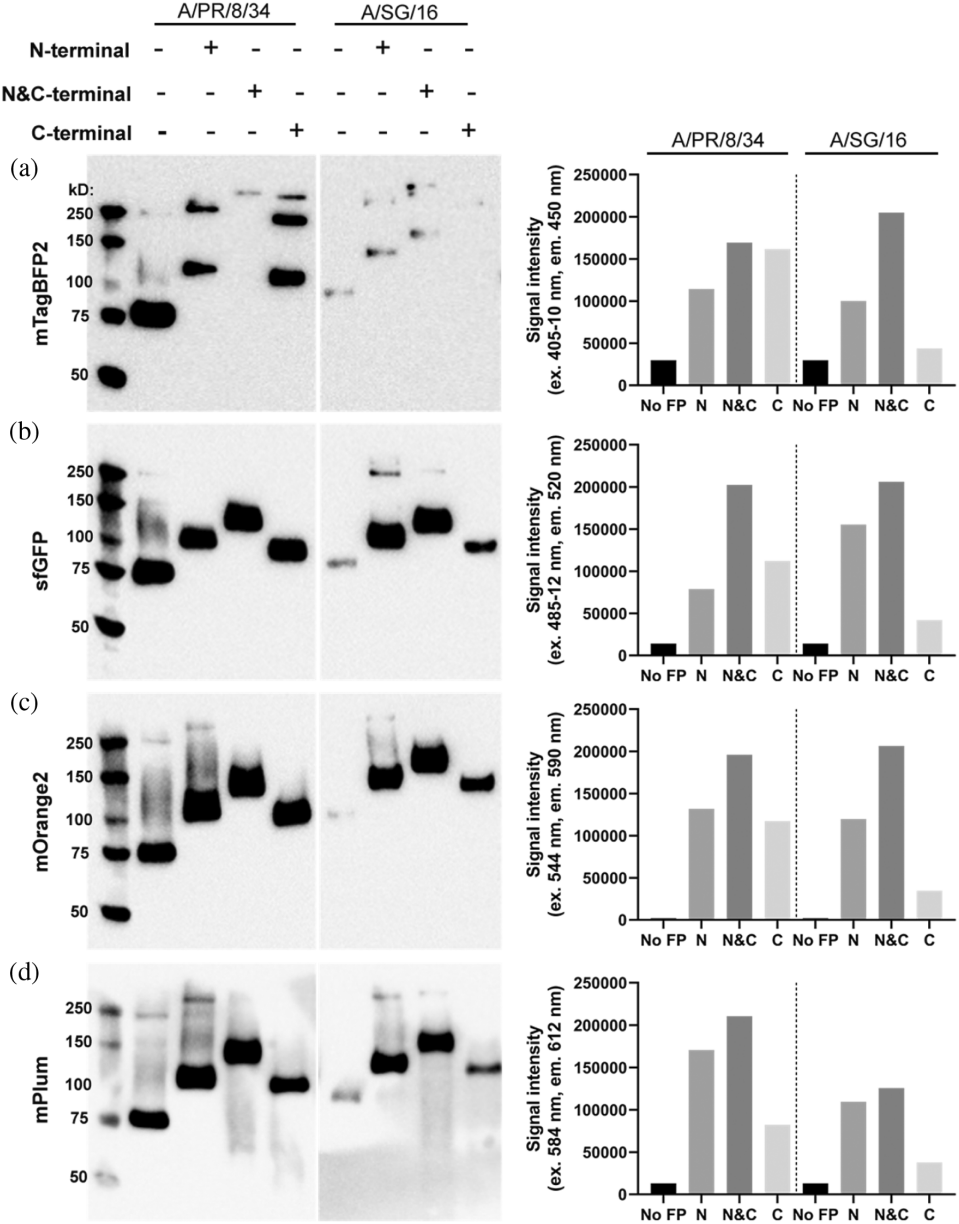
Expression of HA‐fusions with fluorescent proteins characterized using biochemical assays. A/PR/8/34 and A/SG/16 HAs expressed with different FFPs (mTagBFP2, sfGFP, mOrange2, mPlum), whereby the FFP was located at the N‐, N&C or C‐terminus. Expression was assessed with Western blot by directly loading the supernatant and functionality of the fluorescent probe was verified with measuring the fluorescence intensities of the supernatant with a fluorescence reader. *N* = 3 for each measurement, performed in triplicate.

To demonstrate the tolerance of N‐terminal fusions using fluorescent proteins, we generated recombinant HAs in an expression plasmid containing A/Puerto Rico/8/1934 (A/PR/8/34) and A/Singapore/INFIMH‐16‐0019/2016 (A/SG/16). A/Puerto Rico/8/1934 is a model strain commonly used as a reference, with A/SG/16 being challenging to express, potentially related to prolonged Golgi retention (Banfield, 2011). We also created the N&C‐terminal fusion and compared both with our previously designed C‐terminal construct (Nemanichvili et al., 2019). The N‐ and N&C‐terminal fusions were well tolerated, as determined by Western blot and direct fluorescence measurement in cell culture supernatants (Figure 2, Figure S1 and Table S1). The mTagBFP2‐fusions displayed an additional protein (aggregate) band on Western blot (Figure 2a), which is not present in the purified mTagBFP2‐fusions characterized with Coomassie stain (Figure S2). Cell culture supernatants, utilized for Western blot and fluorescence measurements, represent the entirety of (un)folded and aggregated fusions; therefore, direct comparison of Western‐blot and fluorescence intensities is not possible for mTagBFP2 (Figure 2a). For mTagBFP2 expression was lower in comparison to sfGFP, mOrange2, and mPlum, as observed with Western blot; however, the mTagBFP2 fusions appeared to be functional since fluorescence signal could be measured. On Western‐blot, the N(&C)‐terminal fusions of mOrange2, mCherry2, and mPlum enhanced the expression of A/PR/8/34 HA in comparison to the C‐terminal and non‐fluorescent control variant, which aligned with the measured fluorescence intensities (Figure 2c,d, Figure S1). For the sfGFP‐fusions of A/PR/8/34 HA (Figure 2b), the expression profiles on Western blot appeared to be similar; however, the fluorescence intensities were highest for the N&C‐terminal followed by the C‐terminal and then the N‐terminal variant. Even though sfGFP‐fusions for A/SG/16 HA and fusions with other fluorescent proteins for both A/PR/8/34 and A/SG/16 HA generally displayed the highest fluorescence intensities for N&C‐terminal, followed by the N‐terminal and then the last C‐terminal fusion. The N‐terminal fusions generally enhanced protein expression. Interestingly, protein expression was negatively influenced for the mTurquoise2 N&C‐terminal fusion of A/PR/8/34 HA, while for A/SG/16 HA, the N(&C)‐terminal mTurquoise2 fusions increased expression. Similarly, for A/SG/16 HA, N(&C)‐terminal fusions with mTagBFP2, sfGFP, mOrange2, mCherry2, and mPlum improved protein expression concerning the C‐terminal fusion and non‐fluorescent HA, with the measured fluorescence intensities matching Western blot expression profiles. Additionally, HA fusions with fluorescent proteins at both ends (mTagBFP2, sfGFP, mOrange2, and mPlum) appeared to display the highest fluorescence signals; however, fluorescence intensities were not doubled. Nonetheless, a fluorescent palette spanning the visible spectrum of recombinantly expressed influenza A HA tolerates FFP at both termini while increasing protein expression yields for a difficult‐to‐express protein (A/SG/16). Furthermore, to assess the applicability of the fluorescent fusion palette, we have designed additional HA strains that require characterization.

### 2.2 Red fluorescent proteins for an improved fluorescent palette

Red‐shifted FFPs are desired for imaging applications due to decreased light scattering in tissues and the separation of autofluorescence (Deliolanis et al., 2008). Thus, we attempted the expression of several additional far‐red fluorescent probes to improve our palette; however, these fluorescent proteins completely abrogated protein expression, or the FFP was no longer fluorescent (Table S2). Several monomeric FFPs reported as monomeric appear to oligomerize (mNeptune, mCardinal, and mKate2) (Wannier et al., 2018), potentially related to the lack of success with our HA fusions. Monomerization of fluorescent proteins is crucial since oligomerization may have adverse effects, such as aggregation and transport, resulting in containment within cellular compartments. Eventually, we managed to express mPlum‐labeled HAs, increasing the expression yield, albeit possessing a lower brightness (Figure 2 and Table S1).

### 2.3 Folding properties of the P.O.I. are not negatively influenced by fluorescent fusion partners

**FIGURE 3:**
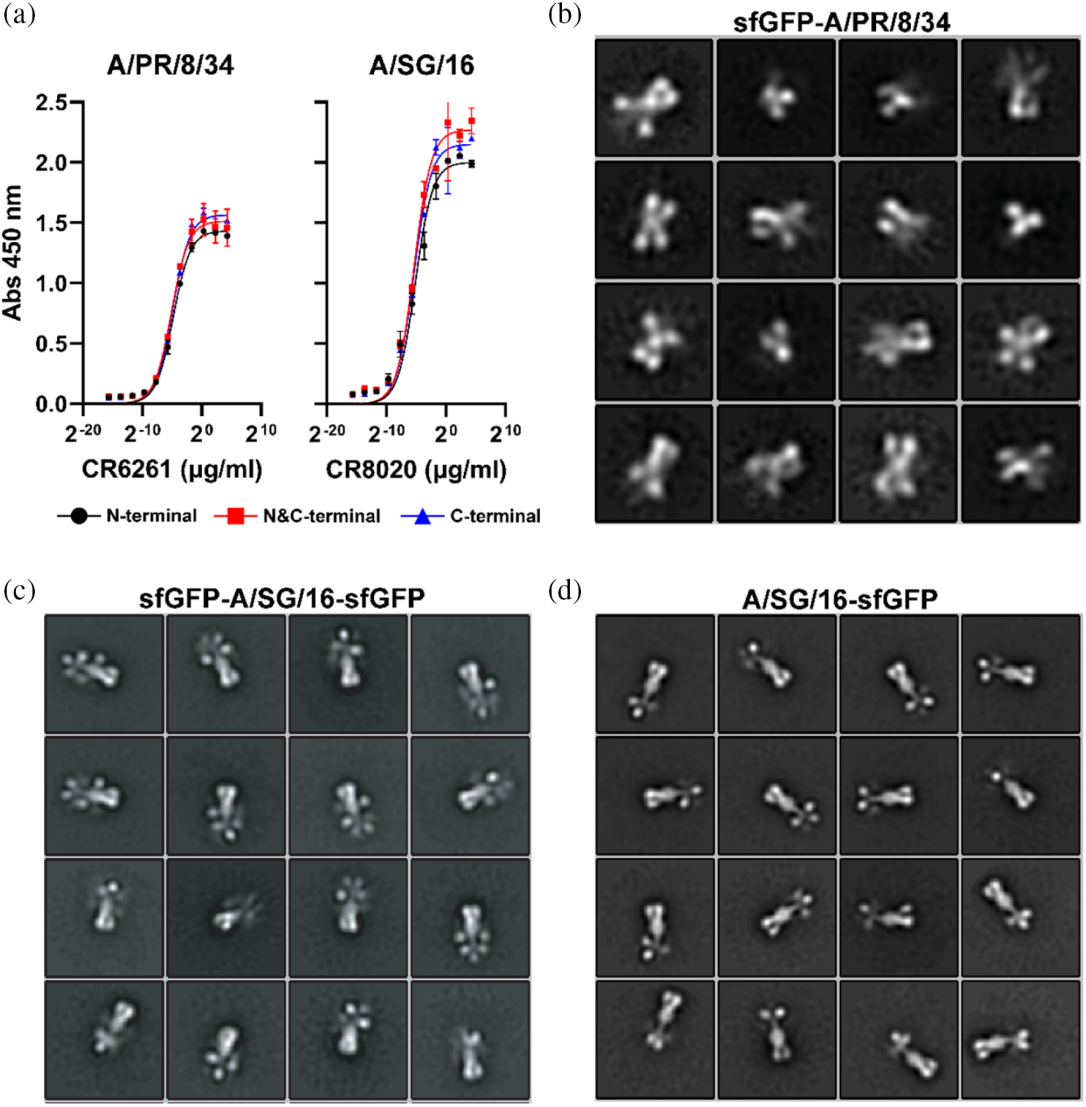
Fluorescent HA fusions are antigenically and structurally similar. (a) ELISA assay to verify antigenicity of fluorescent fusion‐protein (A/PR/8/34 and A/SG/16). Conformation‐dependent stalk antibodies CR6261 and CR8020 utilized to verify conformation and antigenicity of recombinant HA in a concentration‐dependent manner. (b) A/PR/8/34 with an N‐terminal sfGFP folding downwards to the TMD. (c) A/SG/16 with an N&C‐terminal sfGFP, six barrels visibly facing the C‐terminus, therefore tolerating FPs at both ends and folding properly. (d) A/SG/16 with a C‐terminal sfGFP in the tertiary conformation.

A wide palette of fluorescent proteins fused to recombinantly expressed HA appeared functional regarding expression and fluorescence. However, these new fluorescent tools require antigenic and structural verification. Antigenicity was assessed with ELISA assay by utilizing conformation‐dependent stalk‐binding antibodies. Furthermore, to expand on the applicability of the generated fluorescent tools, HA from additional influenza strains was included, and the termini were fused to mOrange2, the best expressing and highly fluorescent fusion protein. For A/PR/8/34 and A/Vietnam/1203/2004 (A/VN/1203/04) stalk antibody CR6261 was utilized (Ekiert et al., 2009), for A/SG/16, A/Hong Kong/8/1968 (A/HK/8/68) and A/duck/Ukraine/1963 (A/duck/UA/63) CR8020 (Tharakaraman et al., 2014). ELISA data shows no difference in antigenicity for each HA strain fused to mOrange2 at the N‐, N&C‐, and C‐termini. Therefore, the fluorescent fusion partner does not influence the folding properties of HAs from distinct subtypes (Figure 3a and Figure S2).

Subsequent characterization was performed with negative stain electron microscopy (ns‐EM) to assess whether the proteins had a native‐like structure and contained three or six barrels. When the sfGFP was present at the N‐ or C‐terminus, three sfGFP barrels were observed, and the protein was native‐like (Figure 3b,d). The sfGFP at the N‐terminus folds downwards towards the C‐terminal region, which directly faces the transmembrane domain (TMD) (Figure 3d) (Nemanichvili et al., 2019). For the N&C‐terminal version, six barrels were observed, and the HA had a native‐like structure (Figure 3c). Thus confirming the tolerance of an N‐terminal FFP when using a transmembrane‐bound protein without influencing the protein folding capacity. Considering the ample distance between the glycan binding domain (HA1) and the location of the N‐ and C‐terminal fusions, binding properties should not be influenced. However, the potential interference of target/receptor binding for its biological functionality also requires assessment.

### 2.4 Receptor binding functionality of HA fluorescent fusion protein

**FIGURE 4:**
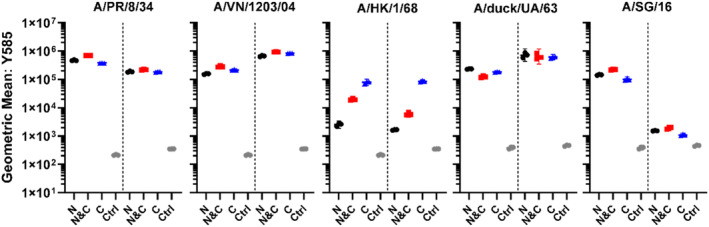
FFP receptor binding functionality. FACS performed with Rajis and MDCKs, in each graph Rajis are depicted on the left and MDCKs on the right. A/PR/8/34, A/VN/1203/4, A/SG/16, and A/duck/UA/63 display receptor binding with minor differences between the N‐, N&C‐ and C‐labeled variants. The presence of three additional mOrange2 barrels did not necessarily increase the fluorescence intensity two‐fold. Ranging from 1.18× to 1.87× difference in fluorescence intensity between N‐ and N&C‐variants and from 1.18× to 2.3× for the C‐ and N&C‐variants. N‐terminal mOrange2 displayed detrimental binding for A/HK/1/68. For A/SG/16 signal is less in MDCK, in relation to Rajis, possibly related to differences in glycan displayed on the cell membrane. Non‐fluorescent HA control = Ctrl. *N* = 3 for each measurement, performed in triplicate.

The FFP does not influence antigenicity and folding properties of recombinantly expressed HA fusions. Therefore, to verify the biological functionality of these fluorescent recombinantly expressed HAs, additional characterization with influenza‐susceptible cell line Madin‐Darby canine kidney (MDCK) (Tsai et al., 2019) and Raji cells was performed. With fluorescence‐activated cell sorting (FACS) binding for A/PR/8/34, A/VN/1203/04, A/HK/1/68, A/SG/16 and A/duck/UA/63 was detected, verifying the biological function of the mOrange2‐HA‐fusions (Figure 4). For A/PR/8/34, A/VN/1203/04, A/duck/UA/63 and A/SG/16 the N&C‐terminally labeled fusions appeared to have a higher signal in comparison to the N‐ or C‐terminal variants. The difference between N‐ and N&C‐variants ranged from 1.18× to 1.87×, while between the C‐ and N&C‐variants ranged from 1.18× to 2.3×. This variance in fluorescence intensity is potentially related to differences in protein maturation and folding of the mOrange2 within the individual HA trimers. Interestingly, A/HK/1/68 displayed different binding properties, dependent on the localization of the fusion protein, with the N‐terminal FFP being detrimental to receptor binding. For A/SG/16, the fluorescence signal was higher for Raji cells than MDCK cells, potentially related to differences in glycan presentation on these cells (Cummings, 2009; Hua et al., 2014; Byrd‐Leotis et al., 2022).

**FIGURE 5:**
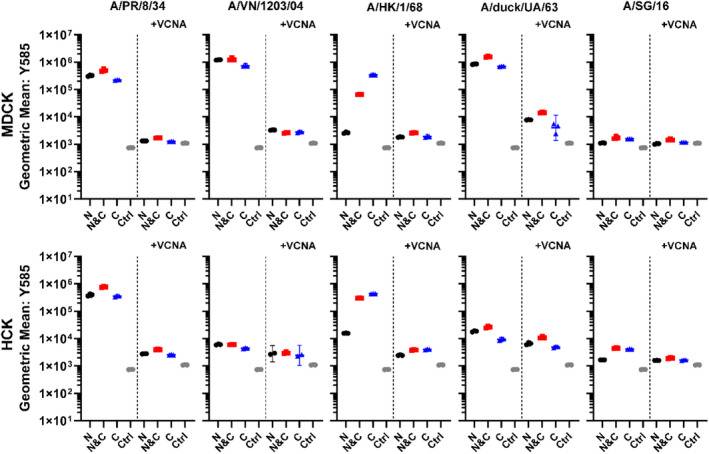
Glycan‐binding functionality of HA‐fusions. FACS performed with MDCK and hCK cells, in each graph untreated cells are depicted on the left and VCNA‐treated cells on the right. A/PR/8/34, A/VN/1203/04, A/HK/1/68, A/SG/16 and A/duck/UA/63 displayed receptor binding on MDCK cells. On hCK cells binding was observed for A/PR/8/34, A/HK/1/68 and A/SG/16. VCNA treatment abrogated binding for all mOrange2‐HA‐fusions on MDCK and hCK cells, displaying that the binding events are SA‐dependent. Non‐fluorescent HA control = Ctrl. *N* = 3 for each measurement, performed in triplicate.

The glycan binding specificity of the mOrange2‐HA‐fusions was further verified with MDCK and humanized canine kidney (hCK) cells (Takada et al., 2019). On MDCK cells, α2‐3 and α2‐6‐linked sialylated glycan structures are naturally presented, while hCK cells display high levels of human receptors (α2‐6‐linked sialylated). Binding for HA A/PR/8/34, A/VN/1203/04, A/HK/1/68, and A/duck/UA/63, with minimal binding for A/SG/16, was observed on MDCK cells (Figure 5). While on hCK cells binding was only observed for HA A/PR/8/34, A/HK/1/68 and A/SG/16. Glycan binding specificities differ between the selected strains and define whether binding occurs on MDCK and hCK cells: A/PR/8/34 (α2‐6‐linked SA), A/VN/1203/04 (α2‐3‐linked SA), A/HK/1/68 (α2‐6‐linked SA), A/SG/16 (α2‐6‐linked SA) and A/duck/UA/63 (α2‐3‐linked SA) (Stevens et al., 2006; Nemanichvili et al., 2019; Canales et al., 2023; Broszeit et al., 2019; Peng et al., 2017, 2018). Following Vibrio cholerae neuraminidase treatment (VCNA, sialidase), binding was abrogated for all mOrange2‐HA‐fusions, demonstrating that the measured binding interactions are SA‐dependent.

### 2.5 Multiparameter characterization of hemagglutinin fusion protein using flow cytometry

**FIGURE 6:**
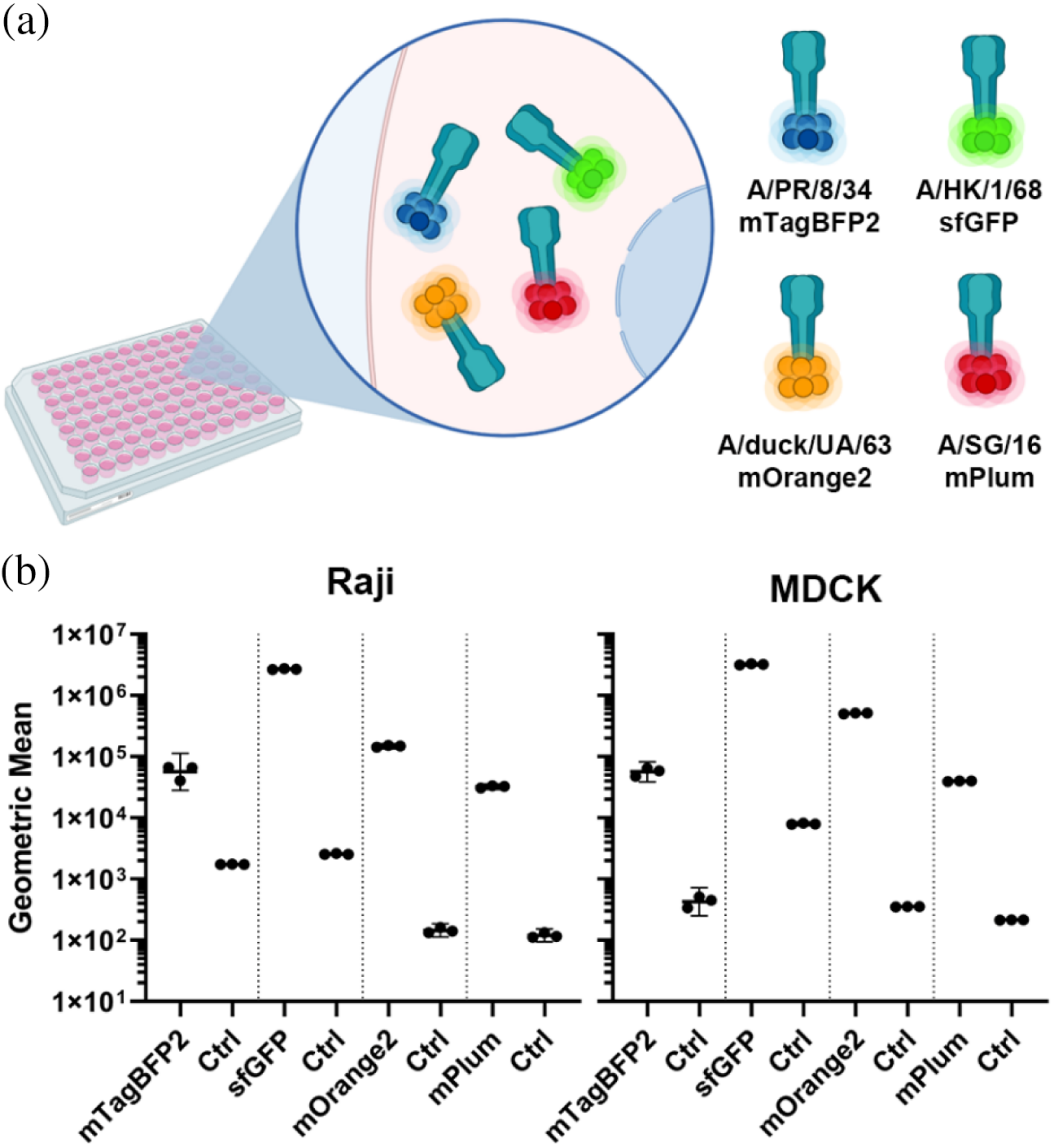
Multiparameter quantification. FACS performed with Rajis and MDCKs, in each graph Rajis are depicted on the left and MDCKs on the right. N&C‐labeled FFP A/PR/8/34‐mTagBFP2, AHK/1/68‐sfGFP, A/duck/UA/63‐mOrange2 and A/SG/16‐mPlum measurements display the possibility for rapid measurement of different receptor‐binding proteins on a certain substrate (cell type. *N* = 3 for each measurement, performed in triplicate. Control (Ctrl).

Multiparameter flow cytometry analysis permits rapid measurement of multiple cellular characteristics of individual cells. Different glycan‐binding HAs that possess distinct receptor specificities can identify and separate cell types within mixed populations based on (co)expressed structures/receptors (O'Donnell et al., 2013). Therefore, a multiparameter experiment was conducted as a proof of concept, using N&C‐labeled FFP A/PR/8/34‐mTagBFP2, A/HK/1/68‐sfGFP, A/duck/UA/63‐mOrange2 and A/SG/16‐mPlum. These HA‐fusions possess distinct spectral properties due to their fluorescent fusion partner, and we assessed whether these fluorescent tools would permit the separation of the spectral channels within the same sample. All HA‐FFPs from different strains, with distinct receptor‐specificities, were first measured individually to generate a compensation matrix for the spectral overlap. Hereafter, A/PR/8/34‐mTagBFP2, A/HK/1/68‐sfGFP, A/duck/UA/63‐mOrange2, and A/SG/16‐mPlum HAs were added simultaneously to either Raji or MDCK cells (Figure 6). Spectral separation of individual HAs fused to distinct FFPs was possible using separate channels. This confirms the possibility of (high‐throughput) multiparameter measurement using HAs with different receptor specificities. Additionally, applying non‐HA proteins would expand on the viability of the designed fluorescent palette for multiparameter measurements.

### 2.6 SARS‐CoV‐2 RBD fluorescent palette multiparameter characterization

**FIGURE 7:**
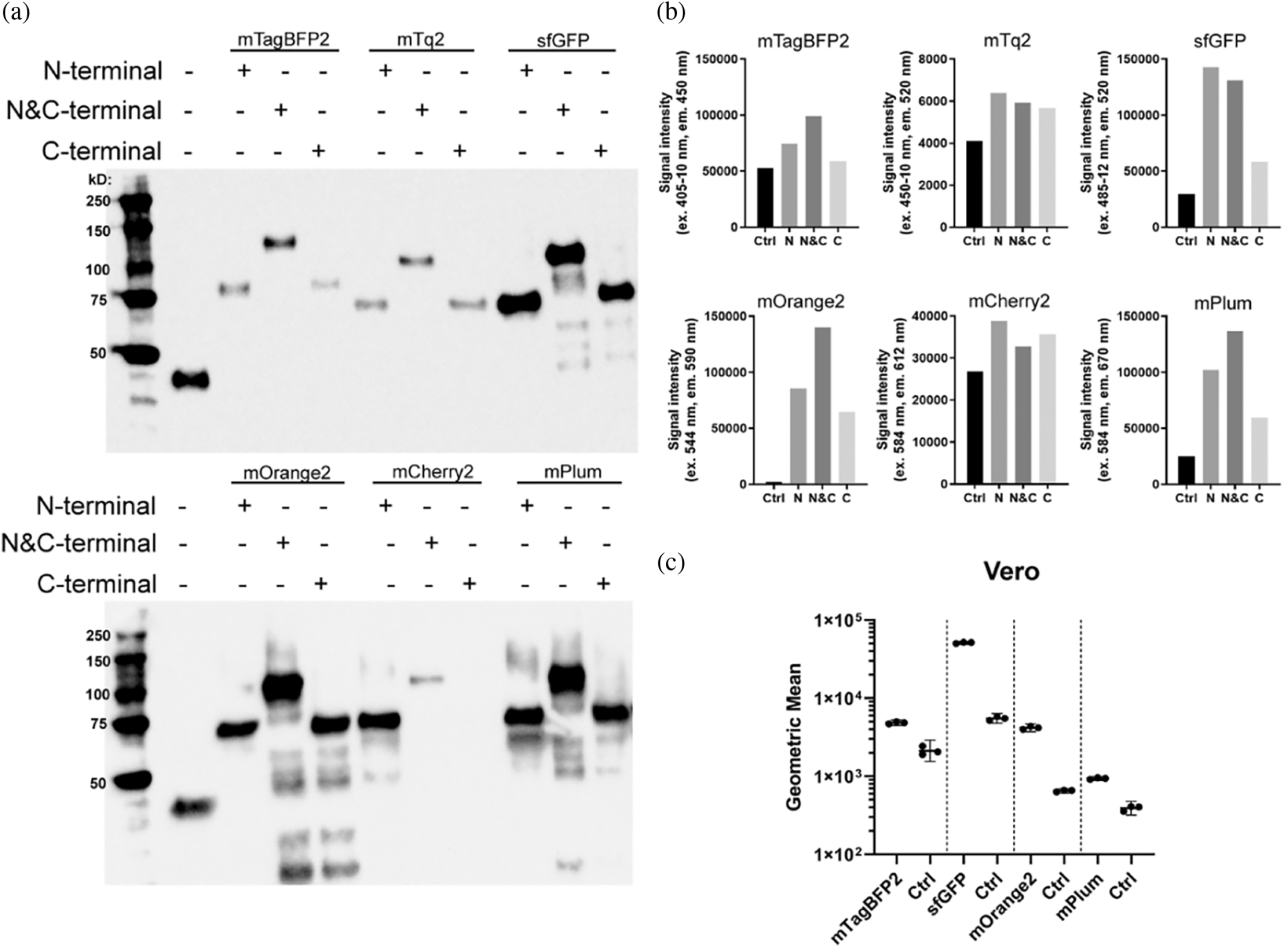
Fluorescent palette of SARS‐CoV‐2 RBD. (a) SARS‐CoV‐2 RBD fused to FFPs (mTagBFP2, mTq2, sfGFP, mOrange2, mCherry2 and mPlum) N‐, N&C‐ and C‐terminally. The N&C labeled variants for all FFPs, except mCherry2, resulted in the highest expression yield. (b) Fluorescence output was measured, the fluorescence signal intensities appeared to overlap with the expression profiles observed with Western blot, except for mTq2 and mCherry2. (c) N&C‐labeled SARS‐CoV‐2 RBD FFP was utilized to assess biological functionality using Vero E6 cells. Control = Ctrl. *N* = 3 for each measurement, performed in triplicate.

To confirm whether our fluorescent tools were viable for other receptor‐binding proteins unrelated to influenza, we replaced the HA open reading frame (ORF) with the ORF of SARS‐CoV‐2 RBD. Similar to HA, differences in expression were observed dependent on the FFP (Figure 7a). The N&C‐terminal variant of mTagBFP2, mTq2, sfGFP mOrange2, and mPlum enhanced the expression concerning the N‐ or C‐labeled proteins. However, only the N‐terminal variant of mCherry2 expression was observed for SARS‐CoV‐2 RBD, but not when fused C‐terminally. SARS‐CoV‐2 RBD expression appeared to decrease in general when fused to mTagBFP2 and mTurqouise2 (mTq2), with higher expression of non‐fluorescent SARS‐CoV‐2 RBD. Furthermore, the measured fluorescence intensities were closer to the background level due to low expression levels of SARS‐CoV‐2 RBD‐mTagBFp2 and ‐mTq2 and fusions (Figure 7b). Incidentally, fusions with sfGFP, mOrange2 and mPlum did improve the expression for all the fusion variants and significant fluorescence was measured (Figure 7b). Biological functionality of these FFPs were verified with Vero E6 cells, which is a relevant cell line for SARS‐CoV‐2 (Ogando et al., 2020). The compensation matrix for HAs was utilized to correct for the spectral overlap. The SARS‐CoV‐2 RBDs labeled with different FFPs appeared to bind efficiently to the Vero E6 cells, and it was possible to separate the individual channels. Like the multi‐color HAs, SARS‐CoV‐2 RBDs displayed the separation power of the differently labeled proteins. Therefore, it demonstrates the functionality of these fluorescent tools for rapid multiparameter measurement using distinct proteins of interest.

## 3 DISCUSSION

Fluorescence techniques have become invaluable tools in the field of biotechnology. Fluorescent proteins have significant advantages compared to synthetic dyes, as the genetic introduction enables imaging of live cells, organelles, and single molecules and monitoring dynamic interactions with proteins or glycans (Nemanichvili et al., 2019; Stepanenko et al., 2013). Fluorescent proteins have different advantages and limitations; thus, a proper selection is necessary to meet experimental requirements. The selection of a fluorescent protein is based on certain crucial factors, such as spectral properties, quantum yield, brightness, maturation rate, fluorescence lifetime, monomeric character, and fidelity in fusions (Chudakov et al., 2010; Snapp, 2005; Kleeman et al., 2018; Cranfill et al., 2016; Thorn, 2017).

Here, we created a set of fluorescent tools that span the visible spectrum using mTagBFP2, mTq2, sfGFP, mOrange2, mCherry2, and mPlum. N‐ and C‐terminal fluorescent fusions are tolerated for HA strains and SARS‐CoV‐2 RBD. The genetic fusions with HA and SARS‐CoV‐2 RBD did not influence the fluorescence functionality of the fluorescent proteins. Furthermore, protein folding and antigenicity were retained, and verification was performed with conformation‐dependent antibodies CR6261/CR8020 and ns‐EM. These antibodies possess the capacity to broadly neutralize multiple influenza subtypes from group 1 (CR6261) and group 2 (CR8020) by targeting highly conserved regions in HA1/HA2 (Joyce et al., 2016). Interestingly, escape mutants are mentioned for H3N2 and H7N7 when using the CR8020 antibody (Ekiert et al., 2011); however, we did not observe lowered sensitivity in ELISA for A/HK/1/68, A/duck/UA/63 and A/SG/16.

The FFPs mTagBFP2 and mTq2 are the brightest and most photostable blue/cyan dyes (Goedhart et al., 2012; Subach et al., 2011), with sfGFP showing high brightness and folding efficiency (Pedelacq et al., 2006). In the orange‐red spectrum, mOrange2 (Shaner et al., 2008) has high photostability, and mCherry2 (Katayama et al., 2008) is a fluorescent protein widely used in live‐cell imaging for its high brightness, stability, monomeric nature, and fast maturation. Specific fusion variants (A/PR/8/34, A/SG/16, and SARS‐CoV‐2 RBD) with mCherry2 displayed lower expression in comparison to mOrange2 and sfGFP fusions, potentially related to previous reports of protein aggregation (Katayama et al., 2008). Therefore, we expanded our palette with mPlum, a similar excitation wavelength as mCherry2 but with a longer emission wavelength for greater tissue penetration (Nguyen et al., 2022; Wang et al., 2004). The expression yields of HA and SARS‐CoV‐2 RBD were enhanced, depending on the FFP and location of the fusion. However, with mTagBFP2 and mTq2 fusions, the overall expression yield was generally lower. Furthermore, mTagBFP2‐fusions contained additional protein species when direct supernatant was utilized for characterization with Western blot; however, these additional bands were not visible for purified mTagBFP2‐fusions. Nonetheless, the mTagBFP2‐fusions of A/PR/8/34 HA and SARS‐CoV‐2 RBD appeared functional during multiparameter flow cytometry analysis. Fusions with sfGFP, mOrange2, and mPlum enhanced expression of HA and SARS‐CoV‐2 RBD concerning the non‐fluorescent control, with the N&C‐fusions outperforming the N‐ and C‐terminal fusions in both expression yield and fluorescence. Furthermore, the N‐terminal fusions were the second‐best performers in expression yield and fluorescence signal, followed by the C‐terminal variants.

The applicability of our fluorescent probes was first expanded and characterized with additional HA from influenza strains A/PR/8/34, A/VN/1203/04, A/HK/1/68, A/SG/16 and A/duck/UA/63. We only utilized the mOrange2 variant to simplify our experimental analysis since this was the FFP with the highest expression yields. The HA‐fusions retained their binding capacity, with the highest fluorescence intensities observed for the N&C‐terminal variants. Interestingly, only for A/HK/1/68 did the N‐terminal fusion appear detrimental to receptor binding. Additionally, sialidase treatment was performed to display that the HA‐fusions mediate all the binding interactions we observed, not non‐specific binding facilitated by the fluorescent protein. Hereafter, for multiparameter analysis, HAs or SARS‐CoV‐2 RBDs were fused to mTagBFP2, sfGFP, mOrange2, and mPlum, with characterization being performed using flow cytometry. Spectral separation of each FFP was possible, demonstrating the applicability of these fluorescent tools for rapid multiparameter measurement.

Fluorescent proteins are oligomeric, wild‐type GFP from *A. Victoria* is part of a heterotetrameric complex (Kremers et al., 2011), and coral and anemone fluorescent proteins are tetrameric (Bindels et al., 2017). Specific mutations have been introduced into these fluorescent proteins to eliminate the oligomerization, which has been successful for jellyfish‐derived fluorescent proteins but not entirely for coral‐derived proteins (Wannier et al., 2018). During the expression of fluorescent proteins, specific problems may occur, such as weak signal, suppression of fluorescence, protein aggregation, incorrect localization, and non‐functional FFP (Cranfill et al., 2016). Oligomeric tendencies of FPs can be evaluated with an organized smooth endoplasmic reticulum (OSER) assay, whereby oligomerization leads to a reconfiguration of the ER (Cranfill et al., 2016; Costantini et al., 2012). Interestingly, mTagBFP2 and sfGFP also display an oligomeric nature; however, we could easily express and utilize these proteins in our experiments. Additionally, the expression of most RFPs was unsuccessful; however, we managed to obtain a functional probe, albeit with low brightness. FFP performance in fusions depends on more than oligomerization, potentially influenced by each fluorescent protein, cell type, mRNA stability, translation efficiency, protein stability, or maturation‐dependent hydrogen peroxide production (Cranfill et al., 2016; Shen et al., 2017; Chen et al., 2011).

All in all, here, we display a set of fluorescent tools that span the visible spectrum. We emphasize that the FFP and the fluorescent protein's location (N‐ and C‐terminus) concerning the P.O.I. can influence the expression yield (van der Woude et al., 2020; Li et al., 2022) without hampering folding and receptor binding capacity. Additionally, FFPs serve as an intrinsic fluorescent handle for read‐out without relying on antibodies for detection. Furthermore, we underline the necessity of adequately evaluating current expression systems and how these probes can enhance expression. We demonstrate the functionality of these fluorescent tools for rapid multiparameter measurement, with the possibility of easily swapping the P.O.I and FFP based on experimental requirements (O'Donnell et al., 2013).

## 4 MATERIALS AND METHODS

### 4.1 HA expression plasmid generation

Codon‐optimized HA encoding sequences of A/Puerto Rico/8/34, A/Vietnam/1203/04, A/Hong Kong/1/1968, A/duck/Ukraine/1963 and A/Singapore/INFIMH‐16‐0019/2016 were cloned into the pCD5 expression as described previously (de Vries et al., 2010). Full‐length SARS‐CoV‐2 S spike 2 (GenBank: MN908947.3) encoding open reading frames (A kind gift of Rogier Sanders, Amsterdam Medical Centre, The Netherlands), the RBD subunit (SARS‐2319–541) was amplified using PCR as described previously (Bouwman et al., 2021). Expression vector pCD5 was adapted for N‐ and N&C‐terminal vectors, the signal sequence was followed by a fluorescent protein open reading frame, the HA‐encoding cDNA, GCN4‐pII trimerization motif (KQIEDKIEEIESKQKKIENEIARIKK), a TEV cleavage site, a fluorescent reporter (N&C vectors) and a Strep‐tag II (WSHPQFEKGGGSGGGSWSHPQFEK; IBA, Germany). The C‐terminal vectors were adapted as described previously (Nemanichvili et al., 2019). The fluorescent reporter open‐reading frames (Table S3) were ordered from Addgene and cloned into the accepting vectors utilizing PCR and Gibson assembly. The mTurquoise2 open reading frame was provided by Joachim Goedhart.

### 4.2 Protein expression and purification

pCD5‐HA‐ +/− GCN4‐fluorescent probe expression vectors were transfected into HEK293S GNT1(−) cells (which are modified HEK293S cells lacking glucosaminyltransferase I activity (ATCC® CRL‐3022™)) with polyethyleneimine I (PEI) in a 1:8 ratio (μg DNA:μg PEI) as previously described (de Vries et al., 2010). The transfection mix was replaced after 6 h by 293 SFM II suspension medium (Invitrogen, 11686029, supplemented with glucose 2.0 g/L, sodium bicarbonate 3.6 g/L, primatone 3.0 g/L (Kerry), 1% glutaMAX (Gibco), 1.5% DMSO and 2 mM valproic acid). For comparison of HA expression levels of all fusion variants, 6‐well plates were transfected, and culture supernatants were harvested 5 days post‐transfection. To compare and analyze the fluorescent fusion proteins with the non‐fluorescent fusion protein control, 10 μL of direct supernatant was loaded onto the SDS‐PAGE gel. Followed by Western‐blot on PVDF membrane (Biorad) using α‐strep‐tag‐HRP mouse antibodies 1:3000 (IBA Life Sciences) with Clarity Western ECL substrate (Biorad, #1705060). Additionally, the fluorescence intensities of the supernatants were measured using a filter‐based PolarStar Omega plate reader. To detect the fluorescence signal, respective fluorescence wavelengths for excitation and emission were utilized (Table S4). Subsequently, HA proteins for experimental characterization were purified from transfections performed on 150 mm dishes with Sepharose Strep‐Tactin beads (IBA Life Sciences) as previously described (de Vries et al., 2010). Proteins were stored in 100 mM Tris and 150 mM NaCl buffer at 4°C. SARS‐CoV‐2 RBD fluorescent fusion protein expression levels were characterized similarly to HA; however, HEK293T cells were utilized instead, as previously described (Bouwman et al., 2021).

### 4.3 Antigenicity of HA proteins

To assess the antigenicity of the HA proteins, 5 μg/mL of HAs with a mOrange2 fluorescent reporter was coated on MaxiSorp 96‐wells plates overnight at 4°C using PBS. Plates were blocked for 3 h with 3% BSA in PBS 0.1% Tween20. CR6261 and CR8020 antibodies were serially diluted 1:1 with a starting concentration of 20 μg/mL, followed by an incubation of 1 h at room temperature. A secondary antibody goat‐anti‐human HRP (31410, Thermo Scientific) at 1:2000 dilution was incubated at room temperature for 1 h to detect the primary antibody. TMB substrate solution (34028, Thermo Scientific) was utilized to develop the plates, and the reaction was stopped after 5 min using 2.5 M H_2_SO_4_. The absorbance was measured at 450 nm with a POLARstar Omega plate reader.

### 4.4 Negative stain electron microscopy structural analysis

HA proteins in 100 mM Tris and 150 mM NaCl at 4°C were diluted to 0.025 mg/mL, deposited on 400 mesh copper negative stain grids for 10 s, and doubled stained with 2% uranyl formate for 10 and 30 s. The grids were imaged on a 120 keV Tecnai Spirit or 200 keV Tecnai T20 electron microscope with a LaB6 filament and a 4k × 4k TemCam F416 camera. Micrographs were collected using Legion (Potter et al., 1999) and then uploaded to Appion (Lander et al., 2009). Particles were picked using DoGPicker (Voss et al., 2009) and further 2D classification was performed using Relion 3.0 (Scheres, 2015).

### 4.5 Biological activity and multiparameter flow cytometry

A/PR/8/34, A/VN/1203/04, A/HK/1/1968, A/duck/UA/1963, and A/SG/2016 were utilized in the flow cytometry measurements, whereby only the mOrange2 labeled proteins were used. Fluorescent HA proteins (100 μg/mL) were precomplexed in FACS buffer (PBS, 0.5% BSA and 2 mM EDTA), for avidity effects (Stevens et al., 2006; Nemanichvili et al., 2019; Tomris et al., 2023; Srinivasan et al., 2008), with a primary α‐strep‐tag mouse antibody (50 μg/mL, 2‐1507‐001, IBA Life Sciences), a secondary rabbit‐anti‐mouse‐HRP (25 μg/mL, NB7544, Novus Biologicals) and incubated for 1 h at 4°C. For each single measurement precomplexed proteins were added to either 50,000 Raji, MDCK or hCK cells, followed by a 1 ‐h incubation period at 4°C. Following the staining cells were washed with FACS buffer and spin down at 200 rcf for 5 min. For VCNA treatment, 1 μL of neuraminidase (NEB, P0720S, 50 units) was added to the cells and treated overnight at 37°C in PBS; hereafter cells were washed with FACS buffer and spin down at 200 rcf, and 50,000 cells were subsequently utilized per well. Viability staining was performed with ViaKrome 808 viability dye (C36628, Beckman Coulter) 1:10,000 diluted in FACS buffer for 5 min at 4°C, followed by centrifugation at 200 rcf for 5 min. Flow cytometry experiments were performed on a Cytoflex LX, and fluorescent signal was detected in the Y585 channel. Gating strategies for the cell population, singlets, time, and viable cells were employed (Figure S4), the fluorescent signal intensities were quantified in the Y585 channel and plotted in GraphPad v9, Geometric mean values with a 95% confidence interval. For the multiparameter HA measurements only N&C‐terminally labeled FFP were utilized, A/PR/8/34 (mTagBFP2), A/HK/1/1968 (sfGFP), A/duck/UA/1963 (mOrange2) and A/SG/2016 (mPlum), proteins (100 μg/mL) were individually precomplexed in FACS buffer with a primary α‐strep‐tag mouse antibody (50 μg/mL, IBA Life Sciences), a secondary rabbit–anti‐mouse‐HRP (25 μg/mL) and incubated for 1 h at 4°C. Hereafter, the HAs with different fluorescent reporters were first used for single stains for the compensation matrix of the flow cytometer, followed by a simultaneous stain with all HAs with different reporters. Fluorescence signal was measured in the V450, B525, Y585 and Y675 channel of the CytoFlex flow cytometer. Gating strategies for the cell population, singlets, time, and viable cells were employed, and the fluorescent signal intensities were plotted in GraphPad v9. Experiments with SARS‐CoV‐2 RBD (100 μg/mL) were performed similarly as the HAs, but without any prior precomplexation. The flow cytometry experiments for SARS‐CoV‐2 RBDs were performed with Vero E6 cells.

## AUTHOR CONTRIBUTIONS

**Ilhan Tomris:** Conceptualization; investigation; formal analysis; data curation; validation; writing – review and editing; writing – original draft. **Roosmarijn van der Woude:** Data curation; investigation; writing – review and editing. **Rebeca de Paiva Froes Rocha:** Software; data curation; visualization. **Alba Torrents de la Peña:** Software; data curation; visualization; writing – review and editing. **Andrew B Ward:** Supervision; resources. **Robert P. de Vries:** Conceptualization; investigation; funding acquisition; project administration; writing – review and editing; supervision; resources.

## Supporting information


**Appendix S1:** Supporting Information.
